# Modulation of Polar Lipid Profiles in *Chlorella* sp. in Response to Nutrient Limitation

**DOI:** 10.3390/metabo9030039

**Published:** 2019-02-28

**Authors:** Daniel A. White, Paul A. Rooks, Susan Kimmance, Karen Tait, Mark Jones, Glen A. Tarran, Charlotte Cook, Carole A. Llewellyn

**Affiliations:** 1 Plymouth Marine Laboratory, Prospect Place, The Hoe, Plymouth, Devon PL1 3DH, UK; paok@pml.ac.uk (P.A.R.); SUKIM@pml.ac.uk (S.K.); KTAIT@pml.ac.uk (K.T.); mark2@pml.ac.uk (M.J.); gat@pml.ac.uk (G.A.T.); chc@pml.ac.uk (C.C.); 2 Department of Biosciences, Singleton Park, Swansea University, Swansea, Wales SA2 8PP, UK

**Keywords:** polar lipids, *Chlorella* sp., LC-MS, nutrient limitation

## Abstract

We evaluate the effects of nutrient limitation on cellular composition of polar lipid classes/species in *Chlorella* sp. using modern polar lipidomic profiling methods (liquid chromatography–tandem mass spectrometry; LC-MS/MS). Total polar lipid concentration was highest in nutrient-replete (HN) cultures with a significant reduction in monogalactosyldiacylglycerol (MGDG), phosphatidylglycerol (PG), phosphatidylcholine (PC), and phosphatidylethanolamine (PE) class concentrations for nutrient-deplete (LN) cultures. Moreover, reductions in the abundance of MGDG relative to total polar lipids versus an increase in the relative abundance of digalactosyldiacylglycerol (DGDG) were recorded in LN cultures. In HN cultures, polar lipid species composition remained relatively constant throughout culture with high degrees of unsaturation associated with acyl moieties. Conversely, in LN cultures lipid species composition shifted towards greater saturation of acyl moieties. Multivariate analyses revealed that changes in the abundance of a number of species contributed to the dissimilarity between LN and HN cultures but with dominant effects from certain species, e.g., reduction in MGDG 34:7 (18:3/16:4). Results demonstrate that *Chlorella* sp. significantly alters its polar lipidome in response to nutrient limitation, and this is discussed in terms of physiological significance and polar lipids production for applied microalgal production systems.

## 1. Introduction

Lipids (e.g., triacylglycerols, wax esters, sterol esters, and polar diacylglycerols) are important constituents of microalgal cells with a broad range of cellular functions including energy storage, membrane structure and integrity, photosynthesis, metabolism, and cell–cell signaling [1,2,3].

In actively growing microalgae, the majority of the fatty acids produced are constituents of polar diacylglycerols (polar lipids), including different classes of glycolipids (monogalactosyldiacylglycerol (MGDG); digalactosyldiacylglycerol (DGDG); sulfoquinovosyldiacylglycerol (SQDG)), phospholipids (phosphatidylcholine (PC), phosphatidylglycerol (PG), phosphatidylethanolamine (PE)), and betaine lipids (e.g., diacylglyceryl trimethyl homoserine (DGTS); see Figure S1 for different polar lipid structures). In green algae, the glycolipids are predominantly composed of 16- and 18-carbon fatty acyl groups which are often polyunsaturated (PUFA), e.g., the omega-3 fatty acid α-linolenic acid (18:3), and are located in thylakoid and chloroplast membranes where they have pivotal roles in photosynthesis, signaling, and regulation [4]. MGDG is usually the most abundant glycolipid (40–55% of plant lipids) compared to DGDG (15–35%) and SQDG (2–40%). Unlike MGDG and DGDG, evidence suggests that SQDG has no specific role in photosynthesis but can act as a substituent for phospholipid under conditions of phosphate limitation [5]. Of the phospholipids, PG is essential for growth and for the photosynthetic transport of electrons and the development of chloroplasts [6]. PE is a nitrogen-containing non-bilayer forming lipid, constituting up to 50% of mitochondrial membrane lipids [7], and has been shown to have an activate role in the xanthophyll cycle [8]. Other than PG, the other lipid classes (PC, PE, betaine lipids) are mainly found in extra chloroplastid membranes within cells. The role of betaine lipids is largely unknown although evidence suggests they can act as a substituent for PC (zwitterionic membrane lipid) in non-PC-producing algae [9]. The composition of fatty acids (different chain lengths and degrees of unsaturation) associated with microalgal lipids varies with growth conditions, including nutrient limitation [10,11,12,13,14,15,16], giving rise to the potential for variation in the number and combination of molecular species contained within each polar lipid class. Therefore, assessment of polar lipid species regulation is essential in elucidating the fundamental lipidomic response and subsequent metabolic regulation within microalgae exposed to different conditions.

In recent years, there has been a growing interest in the applications and bioactivity of polar lipids (especially glycolipids) from algae for use in a range of biotechnological industries [17]. For example, demonstrated glycolipid (MGDG, DGDG, and SQDG) activities include induced apoptosis of human colon carcinoma Caco-2 cells [18], growth inhibition of human melanoma cells and human hepatocellular carcinoma cell lines [19,20], anti-inflammatory activity [21], cholinesterase inhibitory activity [22], activity against protozoans [23], and antibacterial, antiviral, and fungal growth inhibition [24,25,26,27]. The bioactivity of glycolipids has been demonstrated to be related to chemical structure where the sugar molecule and its anomeric configuration, and the carbon length of the acyl chains, are important [28]. The application of phospholipids (irrespective of source) is well recognised, especially in nutraceutical industries, which often take advantage of the amphiphilic nature of these chemicals (see [29]). Since a wealth of information suggests significant biotechnological potential of polar lipids extracted from algae including microalgae, and that the activity of these compounds is influenced by their chemical structure, research is also required to understand the effect of culture conditions on the yield and chemical nature of these compound classes and the species they contain with a view to commercial realisation of microalgal-polar lipid production systems.

The microalga *Chlorella* sp. (Chlorophyceae-green alga) is widely recognised as a useful strain for biotechnology [30], and has been extensively studied under a range of growth conditions, especially nutrient limitation, for the production of triacylglycerol lipids, with a focus on biofuel production [31,32,33,34,35]. Much less is known of the effects of nutrient limitation on the polar lipidome of microalgae, which is important for understanding how microalgae respond to stress by way of lipid metabolism regulation. To better understand these changes, the identification and quantification of polar lipid species can be achieved by using modern liquid chromatography-tandem mass spectrometry (LC-MS/MS) techniques. Indeed, using this approach, polar lipid species profiling has been performed on other microalgae species under different culture conditions, including nutrient stress [36,37,38,39,40,41,42,43]. However, detailed studies concerning the identification and quantification of polar lipid species and their modulation in *Chlorella* sp. under nutrient stress, are surprisingly limited.

In this study, a *Chlorella* sp. isolate was maintained and batch-cultured under different nutrient conditions (nutrient-replete and nutrient-deplete) and was comprehensively evaluated for polar lipid composition and production using a modern mass spectrometry-based approach (LC-MS/MS), to understand how the microalga alters the polar lipidome in response to these environmental conditions. It is envisaged that this fundamental information will be important for understanding the microalgal polar lipidomic response to nutrient deprivation, and for the optimisation of future applied microalgae-polar lipid production technologies and platforms. Findings are discussed in both these contexts.

## 2. Materials and Methods

### 2.1. Microalgal Strain and Culture Conditions

Stock cultures of the *Chlorella* sp. were maintained in the respective experimental media (see below) for a minimum of 6 months with weekly sub-culturing (10% v/v) in fresh media to ensure full physiological adaptation to media nutrient concentrations. All cultures were maintained at 25 °C under 100 µmols photons m^2^ s^−1^ irradiance and 16:8 h light/dark cycle. The culture was originally isolated from an open pond textile factory wastewater in Chennai, India (provided by Dr. Sivasubramanian, Phycospectrum Environment Research Centre, Chennai, India). Species confirmation was provided both taxonomically and by molecular sequencing (GenBank: MF692949.1).

Experimentally, *Chlorella* sp. stock cultures were inoculated (1% v/v) into replicate (*n* = 2) aerated culture flaks (2 L) containing 1 L of either high nutrient-replete (HN) media where nutrients (nitrogen and phosphorous) were known to be replete throughout the culture period, or nutrient-limited media (LN media) where nutrients were known to be exhausted during the culture period (see Table S1 for composition of the media) and grown in batch cultures for up to 16 days. Replicate samples of cultures (typically 10 mL) were taken daily for nutrients, biomass (particulate organic carbon), and cell (*Chlorella* and bacteria) enumeration (see below). Although axenic practices were employed, the *Chlorella* sp. strain used was non-axenic, so bacteria numbers were assessed to determine contribution (based on cellular carbon estimates) to culture biomass and lipid composition. Replicate samples for polar lipid and total fatty acid analyses were taken at different growth stages of the batch cultures: exponential growth phase (Day 4), linear growth phase (Day 9), and late linear or stationary phase (depending on treatment, Days 14–15; see below).

### 2.2. Bacterial Cell Enumeration (Flow Cytometry)

Samples of culture (1 mL) were taken and fixed with 50 µl of 50% glutaraldehyde and stored at −80 °C until analysis. After thawing, the samples were stained with the DNA stain SYBR green (Fisher Scientific, Leicestershire, UK) for 1 h and then analysed using a FACSort flow cytometer (Becton Dickinson, Oxford, UK). Flow cytometer flow rate was calibrated (ca. 11 µL min^−1^) and samples were diluted if required to maintain counts below 1000 events s^−1^.

### 2.3. Particulate Organic Carbon (POC) and Nitrogen Analyses

Cellular material was harvested from cultures by filtration of accurately measured culture volume (typically 5–10 mL) onto ashed glass fiber filters (Whatman GF/F, 25 mm), dried at 60 °C and acidified by fuming with hydrochloric acid prior to analysis. All carbon and nitrogen analyses were carried out on a Thermoquest FlashEA 1112 elemental analyser. Lipid concentrations in cultures (mg mL^−1^; see below) were subsequently normalised to culture biomass (mg C mL^−1^) based on the culture volume from which lipid extracts were generated.

### 2.4. Nutrient Analyses

For media nutrient analyses, 10 mL samples of culture were membrane-filtered (0.2 µm) and the filtrate stored (−20 °C) in an acid-washed bottle to await analysis. After thawing, samples were analysed for nitrate and phosphate concentrations using a nutrient autoanalyser (Branne and Luebbe, AAIII, SPX Flow Technology Ltd., Brixworth, Northampton, UK) using standard methods [44,45].

### 2.5. Lipid Extraction

Culture samples (50 mL) were filtered under light vacuum onto ashed glass fiber filters (Whatman GF/F; 47 mm; *n* = 3) and then stored at −80 °C prior to analyses. Lipid extracts were generated using chloroform/methanol (2:1) and sonication to disrupt cellular material [46]. Samples were further extracted with 100% chloroform to ensure complete lipid extraction. Lipid-containing layers were pooled, dried under vacuum, and stored at −80 °C in 1 mL of chloroform/methanol (2:1).

### 2.6. Polar Lipid Analyses (Liquid Chromatography–Electrospray Ionisation Mass Spectrometry (LC-ESI-MS/MS))

Synthetic phospholipid (dipalmitoyl-PG, -PE, -PC) and betaine lipid (DGTS) standards were purchased from Avanti Lipids Inc. (Alabaster, AL, USA). Purified natural glycolipid standards (MGDG, DGDG, and SQDG) were purchased from Lipid Products (South Nutfield, UK).

Polar lipid standards or aliquots (400 µL) of dried (under N_2_) lipid extract (see above) were re-suspended in dichloromethane/methanol (9:1 v/v; 100 µL) and analysed by LC-ESI-MS as previously described [47]. Samples were injected (2 µL) into an Agilent 1200 LC system (Agilent Technologies UK Ltd., Stockport, Cheshire, UK), and polar lipid classes were chromatographically separated on a 150 × 2.1 mm i.d., 5 µm diol particle size column (PrincetonSpher: Princeton Chromatography Inc., Cranbury, NJ, USA) using a gradient of 100% Solvent A to 52% Solvent B over a period of 20 min and to 71.5% B over 5 min, and this was then held for 10 min, prior to 10 min equilibration time at original solvent conditions. Flow rate was 0.4 mL min^−1^ for 40 min and then increased to 1 mL min^−1^ for rapid column equilibration. Solvents were as follows: Solvent A = 800:200:1.0:0.4 n-hexane/isopropanol/formic acid/25% aqueous ammonium hydroxide; Solvent B = 900:100:1.0:0.4 isopropanol/water/formic acid/25% aqueous ammonium hydroxide. The LC system was coupled to an ion trap mass spectrometer (Agilent 6330: Agilent Technologies UK Ltd., Stockport, Cheshire, UK) with an electrospray ionisation (ESI) source operated in both positive and negative ionisation modes (all samples run separately in each mode). Nitrogen was used as both the nebuliser and drying gas. The MS detector settings were as follows: nebuliser: 35 psi; dry gas flow: 10 L min^−1^; drying temperature: 200 °C; capillary voltage: 4500V; MS/MS fragmentation amplitude: 1.5 V; isolation width: 4 m/z units. Other trap parameters were optimised by infusing standard solutions in isocratic mixtures of Solvents A and B.

Using these conditions under positive ionisation, protonated ions ([M+H]^+^; PE, PC, DGTS) or ammonium adducts ([M+NH_4_]^+^; PG, MGDG, DGDG, SQDG, DGTS) of lipid species were formed, and characteristic class-specific neutral loss or product ions were generated in MS/MS data ([47]; see Table S2). Standard curves were generated from the extracted ion chromatogram of the molecular ion (or sum of molecular ions in the case of the glycolipid standards) in positive ionisation mode. All chromatograms (including standards) were smoothed (3 s cycle width) prior to integration. Linear detection ranges were 5–60 pg on column for the glycerophospholipids, SQDG, DGDG, and DGTS and 5–20 pg for MGDG. Zero values reported for polar lipid concentrations were below these linear limits of detection. Standard polar lipid concentrations were reproducible within 10% error between repeat injections, and a concentration range of standard mixtures was injected every 10 samples to monitor instrument response and ensure linearity.

For lipid extracts, determination of the major molecular ions within each lipid class was based on parent molecular ion, comparative retention times with standards, and confirmation of characteristic neutral loss and product fragmentation ions (headgroup) in MS/MS spectra in positive ionisation mode (see Table S2). A subset of samples was used to determine prominent acyl ion combinations in individual species by confirming acyl fragments in MS/MS spectra in either positive (MGDG, DGDG, SQDG; [RCOO+C_3_H_5_O]^+^ fragments) or negative (PG, PE, PC, DGTS: [RCOO]^−^ fragments) ionisation modes (see Figure S2 for examples). For quantification, extracted ion chromatograms (positive ionisation mode) were generated for each species (molecular ion) and peak areas integrated and quantified based on generated standard curves in the absence of appropriate internal standards (see above). Reported total lipid class concentrations (the sum of polar lipid species concentrations within the respective class) in biomass were subsequently calculated based on the total volume of lipid extract derived for known culture biomass (see particulate organic carbon and lipid extraction sections above).

### 2.7. Fatty Acid Analyses (Gas Chromatography-Mass Spectrometry: GC-MS)

Fatty acid composition was also determined in lipid extracts (no prior separation of lipid classes). 400 µL of lipid extract was dried under a gentle stream of nitrogen before adding nonadecanoic acid (C19:0; 20 µL, 1 mg mL^−1^) as an internal standard. Cellular fatty acids were converted directly to fatty acid methyl esters (FAMEs) by adding 1 mL of transesterification mix (95:5 v/v 3N methanolic hydrochloric acid/2,2-dimethoxypropane) followed by incubation at 90 °C for 2 h [48]. After cooling, FAMEs were recovered by addition of a 1% w/v NaCl solution (1 mL) and n-hexane (1 mL) followed by vortexing. The upper hexane layer was injected directly into the GC-MS system and FAMEs were separated on a fused silica capillary column (30 m × 0.25 mm × 0.25 µm: Omegawax™ 250, Supelco, Sigma-Aldrich, Gillingham, Dorset, UK) using an oven temperature gradient of 75 °C to 240 °C at 4 °C min^−1^ followed by 15 min of hold time. Helium was used as the carrier gas (1 mL min^−1^) and the injector and detector inlet temperatures were maintained at 280 °C and 230 °C, respectively. FAMEs were identified using retention times and qualifier ion response, and quantified using respective target ion responses. All parameters were derived from calibration curves generated from a FAME standard mix (Supelco, Sigma-Aldrich, Gillingham, Dorset, UK).

### 2.8. Data Analyses

Mean values from replicate cultures within treatment are reported ± variance. Comparison of biomass and lipid concentrations between treatments was conducted across the whole batch culture period using t-tests using the software package Minitab 17 (Minitab Ltd., Brandon Court, Coventry, UK). Linear Pearson’s correlation coefficients between biomass and lipid were carried out using the same software. Figures were generated using Sigma Plot 12.0 (Systat Software Inc., San Jose, CA, USA).

Polar lipid species data was further analysed using non-parametric multivariate methods in Primer v6 [49,50]. Resemblances among samples were calculated using the Bray–Curtis resemblance measure [50]. Subsequently, the significance of dissimilarity in lipid data between samples was confirmed using ANOSIM (analysis of similarities; [51]) where large positive R values (range from 0 to 1) indicated distinct separation of lipid composition between the treatments. Subsequently, the polar lipid species contributing to the dissimilarity between samples were explored using the SIMPER routine in the same software package [52].

## 3. Results

**Figure 1:**
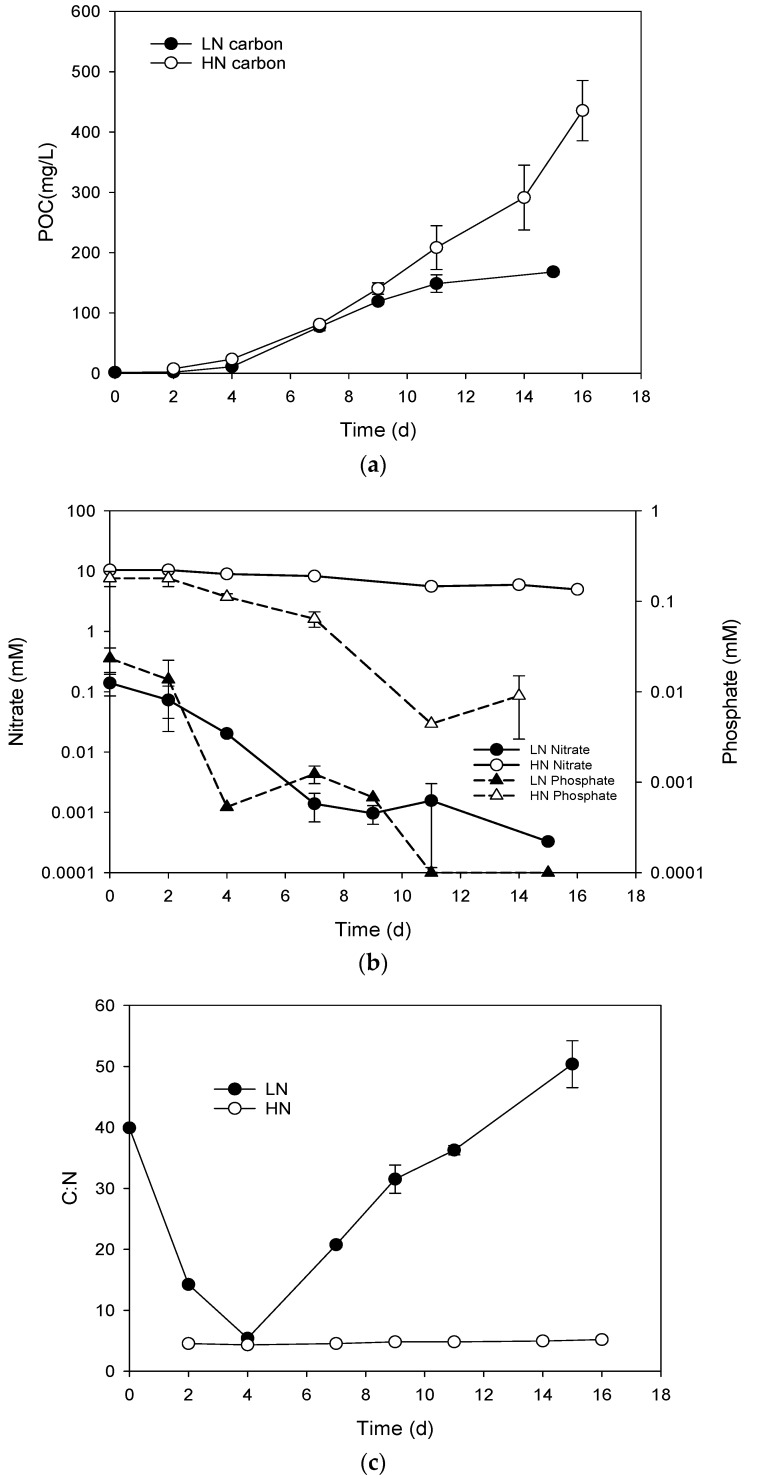
(**a**) Biomass (particulate organic carbon, POC) production, (**b**) media nutrient concentrations, and (**c**) particulate carbon/nitrogen ratio in *Chlorella* sp. grown in batch cultures containing nutrient-limited (LN) or nutrient-replete (HN) media (legends inset).

Biomass measured as POC in both HN and LN media followed typical growth patterns with an initial lag phase (up to ca. 4 days) followed by linear growth in the HN cultures, but with early onset of the stationary phase at ca. 11 days in the LN cultures (Figure 1a).

Biomass in HN cultures at the end of the investigation was more than double (ca. 450 mg C L^−1^) those grown in LN media (ca. 200 mg C L^−1^). Based on 60% carbon composition of dry weight (dw), this equates to approximately 750 mg L^−1^ dw and 333 mg L^−1^ dw in HN and LN cultures, respectively. Biomass productivity within the linear phase of growth across both treatments (up to 9 days of batch culture) was similar at 15.6 ± 1.1 mg C L^−1^ day^−1^ and 13.2 ± 0.5 mg C L^−1^ day^−1^ for the HN and LN treatments, respectively. Biomass contribution from bacteria in cultures (carbon units) was assessed based on monitoring bacterial cell numbers by flow cytometry and estimates of carbon contribution based on 10 fg C cell^−1^ [53]. Across all treatments at any sampling point maximum biomass contribution of bacterial carbon was calculated as <1.6% w/w and was therefore deemed to have insignificant influence on lipid results (data not shown).

Media nitrate concentrations in the HN cultures were not fully depleted during the batch culture and were still high (ca. 5 mM) at the end of the experiment (16 days; Figure 1b). Likewise, phosphate levels were reduced to 0.01 mM but were not fully depleted. Nitrate concentrations in the LN cultures were reduced to 0.0003 mM, and phosphate concentrations were reduced to non-detectable concentrations (<0.0001 mM) at the end of the culture period. The carbon/nitrogen ratio of the biomass stayed consistent at a value of around 5 throughout the culture period in the HN cultures (Figure 1c). In the LN cultures, the carbon/nitrogen ratio increased linearly from 5 near the beginning of the culture (Day 4) to a value of 50 at the end of the culture period.

**Figure 2:**
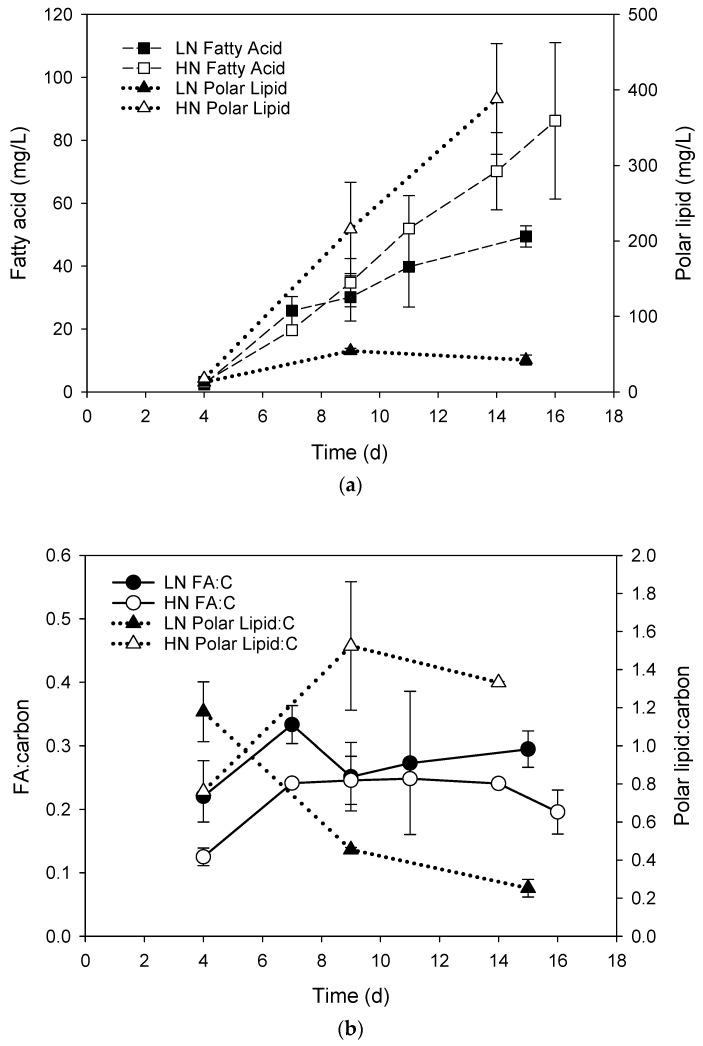
(**a**) Volumetric fatty acid and polar lipid concentrations and (**b**) fatty acid and polar lipid/carbon ratios (mg:mg) in *Chlorella* sp. grown in batch cultures containing LN or HN media (legends inset).

The biomass increase in cultures was reflected in the volumetric concentration of total polar lipids, which was highly correlated with POC (R^2^ = 0.99, *p* < 0.001) in the HN cultures. The relationship between polar lipids and biomass was lower in the LN cultures and was just significant (R^2^ = 0.82, *p* = 0.048). Final concentrations of total polar lipids (Figure 2a) was ca. 10 times higher in the HN cultures compared to the LN cultures (388 ± 73 mg L^−1^ and 42 ± 7 mg L^−1^, respectively) and based on the linear phase of microalgal growth across treatments (up to 9 days) had a significantly higher productivity (24.0 mg L^−1^ day^−1^ and 6.0 ± 0.4 mg L^−1^ day^−1^, respectively). The polar lipid/carbon ratio (Figure 2b) showed an increasing trend in the HN cultures from a value of 0.8 to 1.3 and a decreasing trend in the LN culture from 1.2 to 0.25 at the end of the batch culture and mean values over the course of the experiment were significantly different (*p* = 0.04).

The total fatty acid concentrations of the HN and LN cultures were significantly correlated with biomass (R^2^ = 0.98, *p* < 0.001 and 0.98, *p* = 0.002, respectively). Final concentrations of total fatty acids in the HN and LN cultures were 86.2 ± 24.9 and 49.5 ± 3.4 mg L^−1^, respectively, but with similar productivities (over linear growth phase) of 3.9 ± 0.9 mg L^−1^ day^−1^ and 3.3 ± 0.8 mg L^−1^ day^−1^, respectively. The fatty acid/carbon ratios in both the HN and LN cultures were similar and remained relatively steady throughout the course of the culture period (mean value of 0.22 ± 0.05 and 0.27 ± 0.04, respectively).

**Figure 3:**
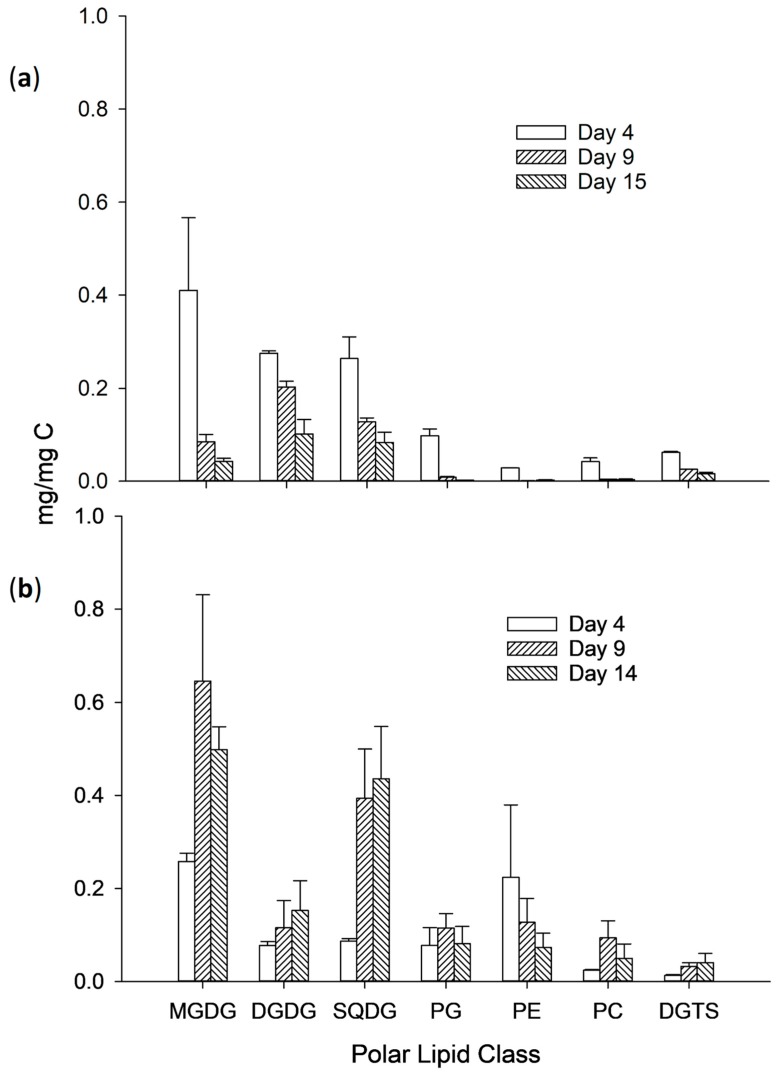
Polar lipid class concentrations in *Chlorella* sp. Biomass grown in batch cultures containing either (**a**) LN or (**b**) HN media (sampling day legend inset). Lipid classes were as follows: monogalactosyldiacylglycerol (MGDG); digalactosyldiacylglycerol (DGDG); sulfoquinovosyldiacylglycerol (SQDG); phosphatidylcholine (PC); phosphatidylglycerol (PG); phosphatidylethanolamine (PE), diacylglyceryl trimethyl homoserine (DGTS).

Closer examination of the polar lipid class concentration normalised for carbon (microalgal biomass; Figure 3) showed that irrespective of culture conditions the glycolipids (MGDG, DGDG and SQDG) were generally more abundant in *Chlorella* lipid extracts than the phospholipids (PG, PE, and PC) and betaine lipid (DGTS). Of these lipid classes, MGDG was the most abundant glycolipid constituting 35–43% consistently in the HN cultures throughout the batch culture but decreasing from 36 to 19% in the LN cultures. Concentrations of MGDG were overall significantly (*p* = 0.025) higher in *Chlorella*-consortia biomass grown in HN media with mean levels of 0.5 ± 0.22 mg mg C^−1^ compared to 0.2 ± 0.2 mg mg C^−1^ in LN cultures. Concentrations of DGDG increased in HN cultures biomass to a maximum of 0.15 ± 0.06 mg mg C^−1^ but relative contribution to total polar lipid remained constant (6–10%). The concentrations of DGDG in LN cultures biomass decreased from a maximum of 0.27 ± 0.01 mg mg C^−1^ to a minimum of 0.10 ± 0.03 mg mg C^−1^ at the end of culture. However, contribution to total polar lipid actually increased from 20 to 37%. Overall mean concentrations of DGDG were not significantly different (*p* = 0.07) between HN and LN cultures. Concentrations of SQDG increased in HN cultures biomass to maximum levels of 0.4 ± 0.1 mg mg C^−1^ and accounted for 11–31% of the total polar lipids. Conversely, concentrations of SQDG fell in LN cultures biomass from 0.3 ± 0.05 to 0.1 ± 0.02 mg mg C^−1^. SQDG accounted for 22 to 34% of total polar lipid in LN cultures. Overall mean concentrations of SQDG across the culture period were not significantly different (*p* = 0.109). The concentrations of PG were relatively constant in the HN cultures biomass (0.08–0.1 mg mg C^−1^ and accounted for 7–11% of polar lipids) but decreased in LN cultures biomass from 0.1 to 0.002 mg mg C^−1^ (dropping from 9 to 1% of total polar lipid). Overall mean concentrations of PG were significantly higher (*p* = 0.043) in the HN cultures. Concentrations of PC constituted between 4 and 7% of polar lipid classes in HN cultures biomass (maximum concentration 0.05 ± 0.03 mg mg C^−1^) and similarly between 2 and 4% in LN cultures (maximum concentration 0.04 mg/mg C) and mean concentrations were overall significantly higher (*p* = 0.047) in the HN cultures. Over the batch culture period, concentrations of PE fell in HN cultures biomass from 0.22 ± 0.16 to 0.07 ± 0.03 mg mg C^−1^ and PE concentrations in LN cultures fell from 0.03 to 0.002 mg mg C^−1^. Overall mean concentrations of PE were significantly higher (*p* = 0.015) in the HN cultures. DGTS concentrations increased throughout the culture in HN culture biomass from 0.01 to 0.04 mg mg C^−1^, but relative contribution to total polar lipids was consistent (ca. 2–4%). However, DGTS concentrations fell in LN cultures biomass (from 0.06 to 0.02 mg mg C^−1^) but again retained consistent levels relative to other polar lipid classes (7–8%). Overall mean concentrations were not significantly different between LN and HN cultures (*p* = 0.59).

**Figure 4:**
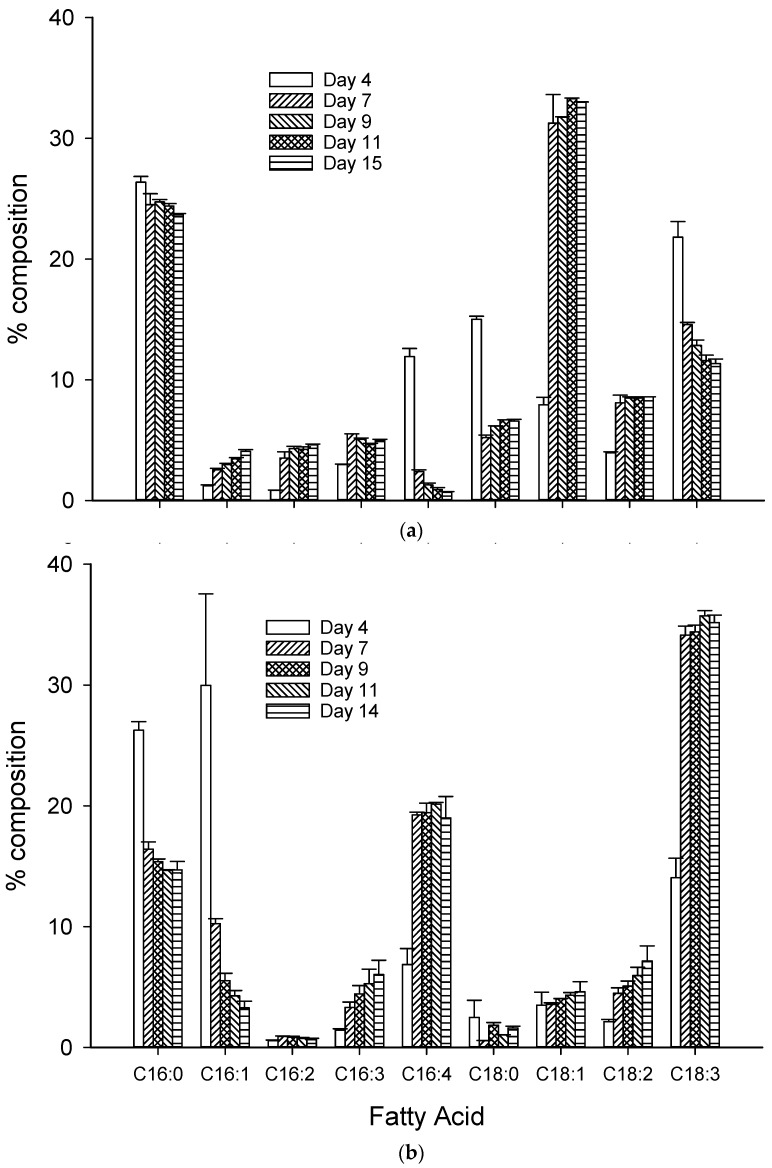
Fatty acid composition (% of total fatty acids) in *Chlorella* sp. in batch cultures containing either (**a**) LN media or (**b**) HN media (sampling day legend inset).

A total of 50 different polar lipid species (6 MGDG, 10 DGDG, 4 SQDG, 6 PG, 9 PE, 8 PC, and 7 DGTS) were identified in *Chlorella* lipid extracts using LC-MS/MS in this study (Table 1). The acyl groups associated with each species mainly constituted combinations of 16 and 18 carbon chains with different degrees of unsaturation (up to 4 double bonds) in agreement with the fatty acid composition of the same lipid extracts (Figure 4). In the HN treatments, the two major (>80% total) MGDG species (acyl carbon:double bonds) were 34:6 (18:3/16:3 and 18:2/16:4) and 34:7 (18:3/16:4). In the LN treatments, the composition of MGDG species varied with the day of the batch culture with notable decreases in MGDG 34:7 (18:3/16:4; from 78% to 2%) and increases in MGDG 34:6 (18:3/16:3; 15–42%), MGDG 34:5 (18:3/16:2; 1% to 28%), and MGDG 34:4 (18:1/16:3; 1–14%) from the start to the end of the batch culture. This is in agreement with the reduction in the fatty acids 16:4 and 18:3 (main acyl groups in MGDG 34:7).

Similarly, DGDG species abundance was comparatively consistent in HN samples taken throughout the batch culture, but in LN cultures there were notable reductions in DGDG 34:7 (18:3/16:4; from 15 to 0%) and subsequent increases in the relative abundance of other DGDG species. The composition of SQDG species was again consistent in the HN cultures irrespective of sampling day but with notable reductions in SQDG 34:3 (18:3/16:0; 45–27%) and increases in SQDG 34:2 (18:2/16:2; 12–17%) and SQDG 34:1 (18:1/16:0; 5–19%) in LN cultures. This observed consistency in the relative composition of species noted for glycolipids in lipid extracts derived from HN *Chlorella* cultures was largely reflected in PC and DGTS classes, as was the decrease in PC and DGTS species, and indeed PG species, containing highly unsaturated acyl moieties in lipid extracts from the LN treatment. However, the relative abundance of PG species in HN extracts was more variable with notable decreases in PG 32:1 (16:0/16:1; from 10 to ca. 3%), PG 34:2 (18:1/16:1 and 18:2/16:0; from 44 to ca. 22%), and PG 34:1 (18:1/16:0; from 28 to ca. 14%) and increases in PG 34:4 (18:3/16:1; from 11 to ca. 32%) and PG 34:3 (18:3/16:0 and 18:2/16:1; from 2 to ca. 23%) between Day 4 and the rest of the batch culture sampling points, although overall this was viewed as an increase in the level of unsaturation of PG species.

**Figure 5:**
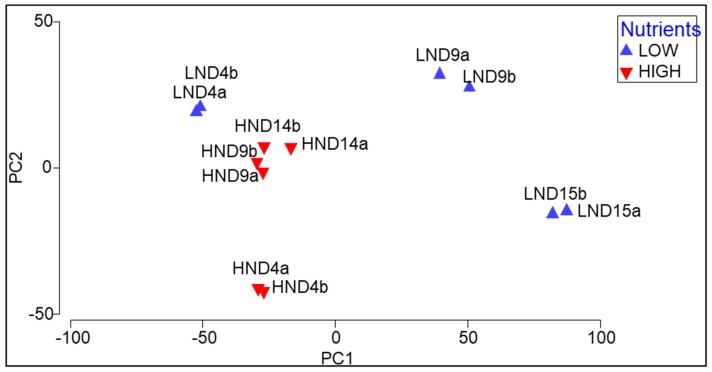
Principal component analyses (PCAs) of samples at different time points (Day = D) of *Chlorella* sp. grown in batch cultures (*n* = 2; a, b) containing LN or HN media based on the polar lipid species profiles (legends inset). PC1 and PC2 explain 73.3 and 17.3% of variation, respectively (cumulative 90.6% of variation explained by the model).

Principal component analyses (PCAs) of the samples from different treatments based on the polar species composition (Figure 5) revealed separation along the PC1 axis (explaining 73.3% of the variation) of samples in the LN treatments based on the sample collection day. Notably, LN samples at Day 4 were clustered with HN samples taken at Days 9 and 14, which as a group were distinctive from LN samples taken at later stages in the batch culture. Some separation of samples along the PC2 axis to a lesser extent (explaining 17.3% of the variation), e.g., Day 4 HN samples, were distinctive from Day 9 and 14 HN samples.

SIMPER analyses of polar lipid species contributing up to 70% of the dissimilarity between samples (see Table S3) revealed that the relative abundance of MGDG 34:7 contributed the most dissimilarity between LN samples taken at the beginning (Day 4) and middle (Day 9; 44% contribution) and at the end (Day 14; 29% contribution) with relative abundance being greater at Day 4 in both comparisons.

## 4. Discussion

Microalgae contain a diverse array of polar lipid structures that have several biological functions, including, importantly, maintaining photosynthesis. Recently there has been growing interest in utilising these polar lipid compounds as novel high-value phytochemical bioactives [54,55], as they often contain high concentrations of omega-3 fatty acids and express a range of activities that make them useful chemicals for the cosmetic, pharmaceutical, and functional food industries. Therefore, detailed research is required to understand the effect of culture conditions on the microalgal polar lipidome, to provide a fundamental understanding of lipid metabolism and regulation and with a view to biorefinery optimisation and commercial realisation of microalgal polar lipid production.

In this study, the productivity of polar lipids in cultures on a titre basis was higher when cultures were nutrient-replete, which was linked with the higher concentrations of biomass production. This is consistent with the fact that cultures initially supplied with lower concentrations of nutrients became nutrient-limited (as demonstrated by the concentrations of nitrate in the media and the carbon/nitrogen ratio in biomass) and as a result produced less total polar lipids for photosynthesis and growth. Coupled with the consistent total concentrations of fatty acids in biomass across the batch culture periods, this strongly supports the theory of physiological diversion of synthesised fatty acids from polar lipids into other neutral lipids, e.g., triacyclglycerols (not analysed here), which is a general metabolic feature of microalgae [56,57]. Indeed, authors have reported an increase in the activity of membrane galactolipid-specific acyl hydrolase in nitrogen-deficient cells of the alga *Dunaliella salina* [58] and *Chlamydomonas reinhardtii* [59], suggesting that liberated fatty acids are available for incorporation into TAGs. This would support the notion that the optimal commercial production of polar lipids in microalgal biomass will require nutrient-intensive culture systems. Therefore, consideration should be given to the cost requirements of nutrient supply versus the yield of bioactive polar lipids from microalgal biomass on this basis, and high nutrient wastewater systems, e.g., municipal and agricultural wastewater, should be considered where applicable [60]. Furthermore, semi-continuous or continuous production systems should be strongly considered over batch culture production, since the former permits a near constant supply of nutrients avoiding nutrient limitation scenarios.

The relative composition of polar lipid classes in lipid extracts from *Chlorella* cultures under nutrient-replete conditions was consistent with the literature where glycolipids dominated over other polar lipid classes in terms of abundance [57]. Glycolipids are principally found in the chloroplasts of eukaryotic algae, notably in thylakoid membranes (especially MGDG and DGDG) where they play critical roles in photosynthesis [4]. Other than PG, the other lipid classes identified are found mainly in extra-chloroplastid membranes.

The notable reduction in the concentrations of MGDG under conditions of nutrient limitation are consistent with the role of this polar lipid class as the major constituent thylakoid lipid and its role in the xanthophyll cycle and photosynthetic pigment-protein complexes [61]. Indeed, MGDG is enriched in chloroplast thylakoids compared to whole cells or envelope membranes in *Chlamydomonas* [9]. DGDG has a role in the structural integrity of Photosystem II, the assembly of the light harvesting complex, and the stability of Photosystem I [61], and it would be reasonable to expect a consistent relative level of reduction in line with MGDG. Interestingly, in this study the significant relative increase in the concentrations of DGDG as a function of total polar lipid (which was not generally consistent with the other polar lipid classes) suggests a physiological stress response adaptation to nutrient stress. These findings are consistent with previous works demonstrating a decrease in the concentrations of MGDG and corresponding increases in the concentrations of DGDG in nitrate-starved *Chlamydomonas nivalis* [62]. This was explained as a biological requirement for the stabilisation of thylakoid membranes in a bi-layer state where fewer proteins and pigments are available under nutrient stress, i.e., an increase of bilayer-forming DGDG and a reduction of hexagonal structures of MGDG. However, it is not possible here to say whether the relative increase in DGDG was a direct result of the substitution of phospholipids or as a result of a greater reduction of other lipid classes generally. It is unclear whether these stress mechanisms can be exploited to produce higher concentrations of DGDG should the bioactivity or biotechnological potential of this glycolipid be more desirable than MGDG. The relative levels of the glycolipid SQDG were consistent between high-nutrient and low-nutrient treatments. Unlike MGDG and DGDG, SQDG is not restricted to photosynthetic membranes.

The phospholipid PG showed a significant reduction in abundance as a function of biomass in nutrient-limited cultures. Since studies have demonstrated that PG is essential for growth of higher plants and cyanobacteria and for the photosynthetic transport of electrons and the development of chloroplasts [6], a reduction under nutrient-limited conditions may be anticipated. However, this is in contrast to the findings of Martin et al. [57], who demonstrated elevated concentrations of PG in both *Chlorella* sp. and *Nannochloropsis* sp. under N-limited conditions, although the absolute amounts remained relatively low. These authors explained an increase in PG with N-deprivation as a means to compensate for the loss of chloroplast glycolipids in order to maintain photosynthetic activity, since PG is found in plastid photosynthetic membranes [63] and has a vital role in photosynthesis. One possible explanation for the differences in these studies may be the substitution of PG with SQDG; both PG and SQDG are both anionic in nature at neutral pH, which has been demonstrated under conditions of phosphate limitation in cyanobacteria and plants [64,65]. In this study, the SQDG/PG molar ratio increased from 2.5 at the beginning of culture to a value of 28 at the end of the culture period in the nutrient-limited cultures. In comparison, the SQDG/PG ratio remained comparatively constant (1.0–4.9) in the nutrient-replete cultures. Phosphate was clearly depleted in the nutrient-limited cultures, and this ratio may suggest a substitution mechanism of SQDG for PG. However, given that a co-limitation of nitrate occurred, it may be that photosynthesis and growth was limited to an extent where substitution of PG with SQDG has no physiological significance. Owing to differences in experimentation and in the absence of comparable data, e.g., media nutrient levels, it is difficult to account for the differences in PG results in this study and those of Martin et al. [57]. Clearly the biosynthesis of PG under nutrient limitation in microalgae should be explored further, especially at the molecular level. Furthermore, since SQDG has been shown to have a range of important bioactivities [20], SQDG vs. PG production in commercially relevant microalgae should be studied, notably where phosphate availability is controlled as a single limiting nutrient.

The phospholipids PE and PC are nitrogen-containing lipids and the recorded reduction in their abundance was consistent with N-limitation in the nutrient-limited cultures [57]. Typically, green algae such as *Chlorella* contain DGTS and PC in interchangeable amounts, notably in response to phosphate limitation, presumably as these two lipids have an equivalent membrane function [9]. Overall, our results did not support the substitution of PC with DGTS under nutrient limitation conditions applied in this study.

Although overall there were reductions of polar lipid classes in biomass under nutrient limitation, there were apparent differences in the magnitude to which individual polar lipid classes were modulated as a function of biomass produced. This suggests that control of supplied nutrients, as a stressor to growth, could be controlled to optimise the relative composition of polar lipid classes depending on the desired class required balanced again reduced overall productivity. Obviously, this necessitates further research regarding the comparative bioactivity of specific classes.

Concerning the acyl composition of polar lipids, in general, MGDG tends to have high contents of polyunsaturated fatty acids, while PG and SQDG are generally more saturated with a high level of palmitate SQDG [9]. Green algae such as *Chlorella* and the Euglenophyta contain MGDG and, to a lesser extent, DGDG, with high amounts of 16:4 and 16:3 acyl groups [66]. This is consistent with the acyl distribution of these polar lipid classes in our cultures grown under nutrient-replete conditions. Under nutrient-limited conditions, the relative composition of FA acyl groups in polar lipids was affected similarly across most polar lipid classes, i.e., increase in the saturation of carbon chains. This was particularly noted in the reduction of the highly abundant MGDG 34:7 species (principally contained 18:3/16:4). The abundance of PG PUFAs (18:3) that was increased in the nutrient-replete cultures increased after 24 h, arguably as a physiological response to improve the incorporation of D1 proteins in the thylakoid membranes for maximum photosynthetic capacity. The reduction in the levels of fatty acid unsaturation generally in microalgae in response to nutrient limitation is well documented and is usually associated with greater deposition in neutral lipid classes [10,16,67]. This study supports the notion that the reduction in the levels of unsaturation is common to both neutral and polar lipid classes when nutrient limitation conditions are present. Since the bioactivity of polar lipids likely depends on chemical structure, which is largely affected by acyl combinations, this may offer the potential for modulating activity of polar lipids produced from microalgal based on nutrient control regimes at the expense of overall productivity. However, in-depth research of structure–activity relationships of individual polar lipid species from different classes is required.

In summary, through culture investigations and in-depth analysis using modern polar lipid species profiling (via LC-MS/MS), we have demonstrated the significant diversity of polar lipids species across all polar lipid classes in *Chlorella* sp. cultures and that the polar lipid productivity, class, and species composition can be modulated in response to nutrient availability. It is envisaged that similar studies exploring culture condition effects (particularly growth stressors) on polar lipid composition will inform our fundamental understanding of how microalgae lipid metabolism functions under changing environmental conditions. These findings, coupled with necessary novel research concerning bioactivity analyses of polar lipid classes and species, will help to realise the potential of a microalgal-polar lipid production platform for various future biotechnology applications.

## Figures and Tables

**Table 1:** Relative abundance (%) of polar lipid species (as a function of total species concentration within class) in *Chlorella* sp. grown in batch cultures containing either LN or HN media. Lipid classes were monogalactosyldiacylglycerol (MGDG); digalactosyldiacylglycerol (DGDG); sulfoquinovosyldiacylglycerol (SQDG); phosphatidylcholine (PC); phosphatidylglycerol (PG); phosphatidylethanolamine (PE), diacylglyceryl trimethyl homoserine (DGTS). The carbon:double bond column refers to the total number of carbon atoms and double bonds of acyl groups attached to the diglyceride moiety and the acyl groups column refers to the dominant fatty acid combinations as detected in MS/MS spectra (see Materials and Methods).

Lipid Class	Carbon: Double Bonds			LN			HN	
		Acyl Groups	Day 4	Day 9	Day 15	Day 4	Day 9	Day 14
MGDG	34:7	18:3/16:4	78.4 ± 0.5	11.1 ± 2.0	1.6 ± 0.5	78.5 ± 0.8	62.5 ± 1.3	58.6 ± 3.7
MGDG	34:6	18:3/16:3, 18:2/16:4	14.6 ± 0.2	39.9 ± 1.7	41.5 ± 2.8	15.0 ± 0.4	24.4 ± 1.1	20.8 ± 1.0
MGDG	34:5	18:3/16:2	0.6 ± 0.9	29.9 ± 3.3	28.2 ± 3.0	1.4 ± 0.1	5.6 ± 0.6	6.4 ± 1.0
MGDG	34:4	18:1/16:3	0.6 ± 0.8	12.0 ± 0.7	13.7 ± 0.8	1.6 ± 0.3	2.3 ± 0.3	3.8 ± 0.5
MGDG	34:3	18:1/16:2	0.0 ± 0.0	6.3 ± 0.8	10.1 ± 0.7	0.0 ± 0.0	0.8 ± 0.0	1.6 ± 0.4
MGDG	36:6	18:3/18:3	5.1 ± 0.7	0.7 ± 1.0	4.2 ± 0.2	3.5 ± 0.7	3.4 ± 0.5	5.8 ± 0.0
DGDG	34:7	18:3/16:4	14.6 ± 1.1	1.4 ± 0.6	0.0 ± 0.0	12.5 ± 0.7	20.1 ± 2.4	12.7 ± 0.9
DGDG	34:6	18:3/16:3	33.4 ± 2.4	22.3 ± 3.2	21.3 ± 0.4	31.3 ± 1.0	17.0 ± 1.4	14.5 ± 0.6
DGDG	34:5	18:2/16:3	10.2 ± 0.0	22.4 ± 1.0	21.7 ± 2.0	11.8 ± 0.5	10.2 ± 0.1	9.4 ± 0.8
DGDG	34:4	18:2/16:2	5.3 ± 0.0	9.0 ± 0.9	10.2 ± 0.2	9.5 ± 1.7	12.4 ± 1.0	10.9 ± 0.8
DGDG	34:3	18:3/16:0	16.1 ± 2.1	22.0 ± 1.4	21.4 ± 0.9	13.7 ± 0.5	11.4 ± 0.6	11.5 ± 1.1
DGDG	34:2	18:2/16:0	7.5 ± 0.8	14.6 ± 0.7	15.2 ± 0.5	8.2 ± 0.3	12.0 ± 0.7	14.9 ± 0.1
DGDG	34:1	18:1/16:0	5.6 ± 0.2	8.4 ± 0.2	10.3 ± 1.4	5.8 ± 0.7	8.7 ± 0.2	8.8 ± 0.5
DGDG	36:6	18:3/18:3	6.0 ± 0.2	0.0 ± 0.0	0.0 ± 0.0	5.4 ± 1.3	5.6 ± 1.3	8.9 ± 0.6
DGDG	36:5	18:3/18:2	1.3 ± 0.1	0.0 ± 0.0	0.0 ± 0.0	1.8 ± 0.5	1.8 ± 0.3	4.7 ± 0.3
DGDG	36:4	18:1/18:3	0.0 ± 0.0	0.0 ± 0.0	0.0 ± 0.0	0.0 ± 0.0	0.8 ± 1.1	3.5 ± 0.2
SQDG	32:0	16:0/16:0	37.5 ± 1.0	38.9 ± 2.5	37.9 ± 2.4	36.2 ± 1.3	32.5 ± 0.9	30.0 ± 3.7
SQDG	34:3	18:3/16:0	45.4 ± 0.2	27.1 ± 2.1	26.5 ± 0.7	46.2 ± 0.4	46.9 ± 3.3	45.5 ± 0.9
SQDG	34:2	18:2/16:2	12.0± 0.0	18.2 ± 0.1	17.0 ± 0.1	12.1 ± 0.2	14.5 ± 2.3	17.4 ± 2.0
SQDG	34:1	18:1/16:0	5.1 ± 1.2	15.8 ± 0.3	18.6 ± 1.8	5.5 ± 1.9	6.1 ± 0.2	7.1 ± 0.8
PG	32:1	16:0/16:1	0.0 ± 0.0	0.0 ± 0.0	0.0 ± 0.0	10.3 ± 0.2	3.2 ± 0.2	1.3 ± 0.1
PG	34:4	18:3/16:1	60.7 ± 0.0	31.3 ± 5.0	0.0 ± 0.0	10.8 ± 3.4	31.2 ± 2.5	33.2 ± 0.9
PG	34:3	18:3/16:0, 18:2/16:1	23.4 ± 0.7	23.5 ± 0.6	0.0 ± 0.0	1.8 ± 2.5	22.3 ± 0.5	24.0 ± 3.7
PG	34:2	18:1/16:1, 18:2/16:0	8.1 ± 1.0	16.5 ± 0.7	23.2 ± 8.0	43.9 ± 0.7	23.2 ± 2.4	21.6 ± 1.0
PG	34:1	18:1/16:0	7.8 ± 0.2	28.7 ± 5.0	76.8 ± 8.0	28.0 ± 0.1	14.8 ± 0.1	13.2 ± 1.4
PG	36:2	18:1/18:1	0.0 ± 0.0	0.0 ± 0.0	0.0 ± 0.0	5.2 ± 0.3	5.3 ± 0.6	6.7 ± 2.2
PE	32:1	16:0/16:1	0.0 ± 0.0	0.0 ± 0.0	0.0 ± 0.0	14.2 ± 0.0	10.8 ± 1.1	10.1 ± 1.7
PE	33:1	16:0/17:1	0.0 ± 0.0	0.0 ± 0.0	0.0 ± 0.0	0.4 ± 0.2	0.8 ± 0.3	1.1 ± 0.0
PE	34:2	18:1/16:1	2.3 ± 0.3	13.3 ± 2.3	3.7 ± 5.2	50.7 ± 2.1	48.3 ± 0.3	47.5 ± 1.0
PE	34:1	18:1/16:0	0.0 ± 0.0	0.0 ± 0.0	0.0 ± 0.0	28.4 ± 0.1	22.5 ± 2.3	21.3 ± 1.6
PE	34:0	18:0/16:0	11.2 ± 1.6	0.0 ± 0.0	17.5 ± 11.0	1.4 ± 2.0	2.5 ± 0.6	2.5 ± 0.0
PE	36:5	18:2/18:3	46.2 ± 0.6	22.0 ± 1.4	6.6 ± 9.3	0.7 ± 0.2	2.9 ± 0.0	1.7 ± 1.1
PE	36:4	18:1/18:3, 18:2/18:2	29.7 ± 1.9	36.6 ± 0.4	27.2 ± 2.6	0.0 ± 0.0	2.2 ± 0.0	1.4 ± 2.0
PE	36:3	18:2/18:1	10.6 ± 0.6	28.0 ± 3.3	29.6 ± 1.1	0.0 ± 0.0	0.4 ± 0.5	0.7 ± 1.0
PE	36:2	18:1/18:1	0.0 ± 0.0	0.0 ± 0.0	15.5 ± 10.6	2.3 ± 0.4	3.3 ± 0.0	5.5 ± 0.1
PC	34:4	18:3/16:1	5.3 ± 0.1	1.8 ± 0.8	1.8 ± 0.0	4.4 ± 0.2	6.5 ± 1.2	5.1 ± 0.0
PC	34:3	18:3/16:0, 18:2/16:1	15.1 ± 0.2	6.7 ± 1.0	6.2 ± 1.7	13.5 ±1.8	9.6 ± 0.2	10.4 ± 0.7
PC	34:2	18:2/16:0, 18:1/16:1	3.5 ± 0.0	10.4 ± 1.7	10.3 ± 0.2	5.3 ± 0.3	3.3 ± 0.3	4.7 ± 0.9
PC	36:6	18:3/18:3	36.1 ± 1.2	15.1 ± 4.3	14.3 ± 2.6	36.4 ± 0.1	36.4 ± 0.1	28.4 ± 6.8
PC	36:5	18:3/18:2	22.8 ± 0.2	26.6 ± 1.3	23.5 ± 4.9	20.7 ± 0.2	20.9 ± 1.5	18.7 ± 0.2
PC	36:4	18:3/18:1	11.6 ± 0.7	18.7 ± 0.1	20.0 ±2.1	11.3 ± 0.5	11.7 ± 0.6	17.0 ± 1.9
PC	36:3	18:2/18:1	4.1 ± 0.2	10.9 ± 2.3	13.1 ± 0.5	4.0 ± 0.6	4.9 ± 0.1	8.9 ± 1.3
PC	36:2	18:1/18:1	1.7 ± 0.2	9.8 ± 0.3	11.1 ± 3.2	4.4 ± 0.8	3.3 ± 0.6	6.7 ± 2.1
DGTS	34:4	18:4/16:0	45.0 ± 0.1	25.7 ± 1.7	24.3 ± 0.8	44.6 ± 2.7	33.1 ± 1.2	31.6 ± 0.5
DGTS	34:3	18:3/16:0	16.3 ± 0.6	23.1 ± 1.0	28.8 ± 1.0	14.4 ± 0.9	11.1 ± 0.2	12.1 ± 1.5
DGTS	34:2	18:2/16:0	1.8 ± 0.0	12.0 ± 2.1	15.0 ± 0.3	0.0 ± 0.0	1.4 ± 1.9	3.5 ± 1.8
DGTS	36:7	18:3/18:4	14.8 ± 0.9	3.8 ± 0.4	2.3 ± 0.1	15.6 ± 0.8	23.7 ± 1.7	19.6 ± 4.6
DGTS	36:6	18:3/18:3	14.1 ± 0.2	9.1 ± 0.8	6.3 ± 0.7	14.8 ± 1.6	16.9 ± 0.3	15.2 ± 0.7
DGTS	36:5	18:3/18:2	6.3 ± 0.6	14.5 ± 0.8	11.3 ± 0.1	7.8 ± 1.0	9.2 ± 0.5	11.0 ± 1.0
DGTS	36:4	18:3/18:1	1.7 ± 0.1	11.4 ± 0.5	12.0 ± 0.2	2.9 ± 0.2	4.6 ± 0.5	6.9 ± 1.5

## Supplementary Materials

The following are available online at https://www.mdpi.com/2218-1989/9/3/39/s1. Figure S1: Chemical structures of different polar lipid classes analysed in this study; Figure S2: MS/MS fragmentation spectra for confirmation of acyl groups in polar lipid species using positive ionisation; Table S1: Nutrient composition in HN and LN media used in *Chlorella* sp. batch cultures; Table S2: Diagnostic neutral loss or product ions in MS/MS spectra used to determine class for lipid species analysed using LC-ESI-MS/MS in positive ionisation mode; Table S3: SIMPER analyses of polar lipid species contributing to the dissimilarity between lipid samples taken from *Chlorella* sp. grown in LN media at day 4, day 9, and days 4 and 15 of batch cultures.
