# Supplementary material for: Modulation of Polar Lipid Profiles in Chlorella sp. in Response to Nutrient Limitation
Source: Metabolites. 2019 Feb 28;9(3):39. doi: 10.3390/metabo9030039 (PMC6468466; doi:10.3390/metabo9030039)
Supplement: Supplementary file 1 [file metabolites-09-00039-s001.pdf]

Table S1 Nutrient composition in nutrient-replete (HN) and nutrient-limited (LN) media used in *Chlorella sp*. batch cultures.

<table>
<tr><th>Nutrient</th><th>HN Media
Concentration (mM)</th><th>LN Media
Concentration (mM)</th></tr>
<tr><td>$NaNO_3$</td><td>17.6</td><td>0.23</td></tr>
<tr><td>K_2HPO_4</td><td>0.23</td><td>0.045</td></tr>
<tr><td>$MgSO_4$.7H2O</td><td colspan="2">0.30</td></tr>
<tr><td>$CaCl_2$.2H2O</td><td colspan="2">0.24</td></tr>
<tr><td>Citric acid</td><td colspan="2">0.03</td></tr>
<tr><td>Ammonium ferric citrate green</td><td colspan="2">~0.03</td></tr>
<tr><td>$EDTANa_2$</td><td colspan="2">2.7×10^{-3}</td></tr>
<tr><td>Na_2CO_3</td><td colspan="2">0.19</td></tr>
<tr><td>Trace metal solution[¥]</td><td colspan="2">1 mL L^{-1}</td></tr>
</table>

[¥]Contains per L: 2.86 g H_3BO_3; 1.81g $MnCl_2.4H_2O$; 0.22 g $ZnSO_4.7H_2O$; 0.39 g $Na_2MoO_4.2H_2O$; 0.08 g $CuSO_4.5H_2O$; 0.05 g $Co(NO_3)2.6H_2O$.

Table S2 Diagnostic neutral loss or product ions in MS/MS spectra used to determine class for lipid species analysed using LC-ESI-MS/MS in positive ionisation mode. See materials and methods section for description of MS conditions.

Lipid Class	Molecular ion	Neutral loss or *product ion* mass	Neutral loss or *product ion* description
MGDG	$[M+NH_4]^+$	179; 197	glycosyl; glycolsyl + H_20
DGDG	$[M+NH_4]^+$	341; 359	glycosyl; glycolsyl + H_20
SQDG	$[M+NH_4]^+$	243; 261	glycosyl; glycolsyl + H_20
PG	$[M+NH_4]^+$	189	phosphoglycerol
PE	$[M+H]^+$	141	phosphoethanolamine
PC	$[M+H]^+$	*184*	*Phosphocholine*
DGTS	$[M+H]^+$	*236*	*Headgroup*

Table S3 SIMPER analyses of polar lipid species contributing to the dissimilarity between lipid samples taken from *Chlorella sp*. grown in nutrient-limited (LN) media at day 4 and day 9 and day 4 and 15 of batch cultures.

**Species**	**LN Day 4**	**LN Day 9**	**Contribution %**	**Cumulative contribution %**
MGDG 34:7	78.4	11.1	43.94	43.94
PG 34:4	60.7	31.3	8.46	52.40
MGDG 34:5	0.6	29.9	8.34	60.75
MGDG 34:6	14.6	39.9	6.20	66.94
PC 36:6	36.1	15.1	4.35	71.30

**Species**	**LN Day 4**	**LN Day 15**	**Contribution %**	**Cumulative contribution %**
MGDG 34:7	78.4	1.62	29.23	29.23
PG 34:1	7.8	76.8	23.74	52.97
PG 34:4	60.7	0	18.25	71.21

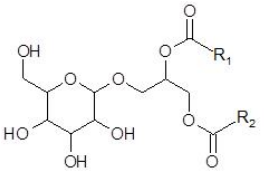

MGDG
monogalatosyldiacylglycerol

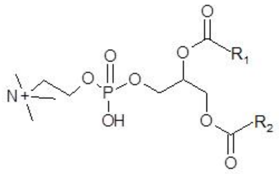

PC
phosphatidylcholine

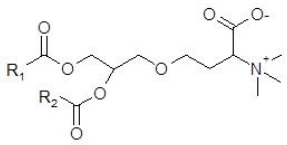

DGTS
Diacylglyceryl-trimethyl-homoserine

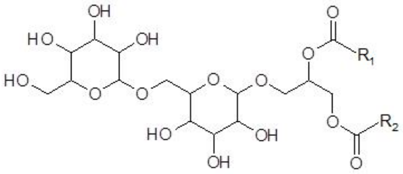

DGDG
digalatosyldiacylglycerol

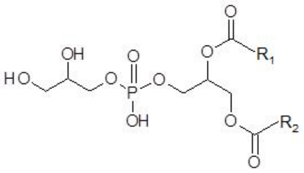

PG
phosphatidylglycerol

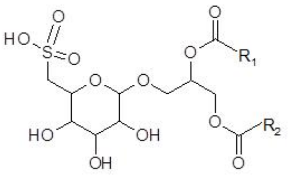

SQDG
sulfoquinovosyldiacylglycerol

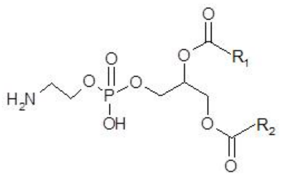

PE
phosphatidylethanolamine

Figure S1 Chemical structures of different polar lipid classes analysed in this study. R_1 and R_2 denote different acyl groups (differeing in carbon chain length and degree of unstauration) that give rise to different species within each class.

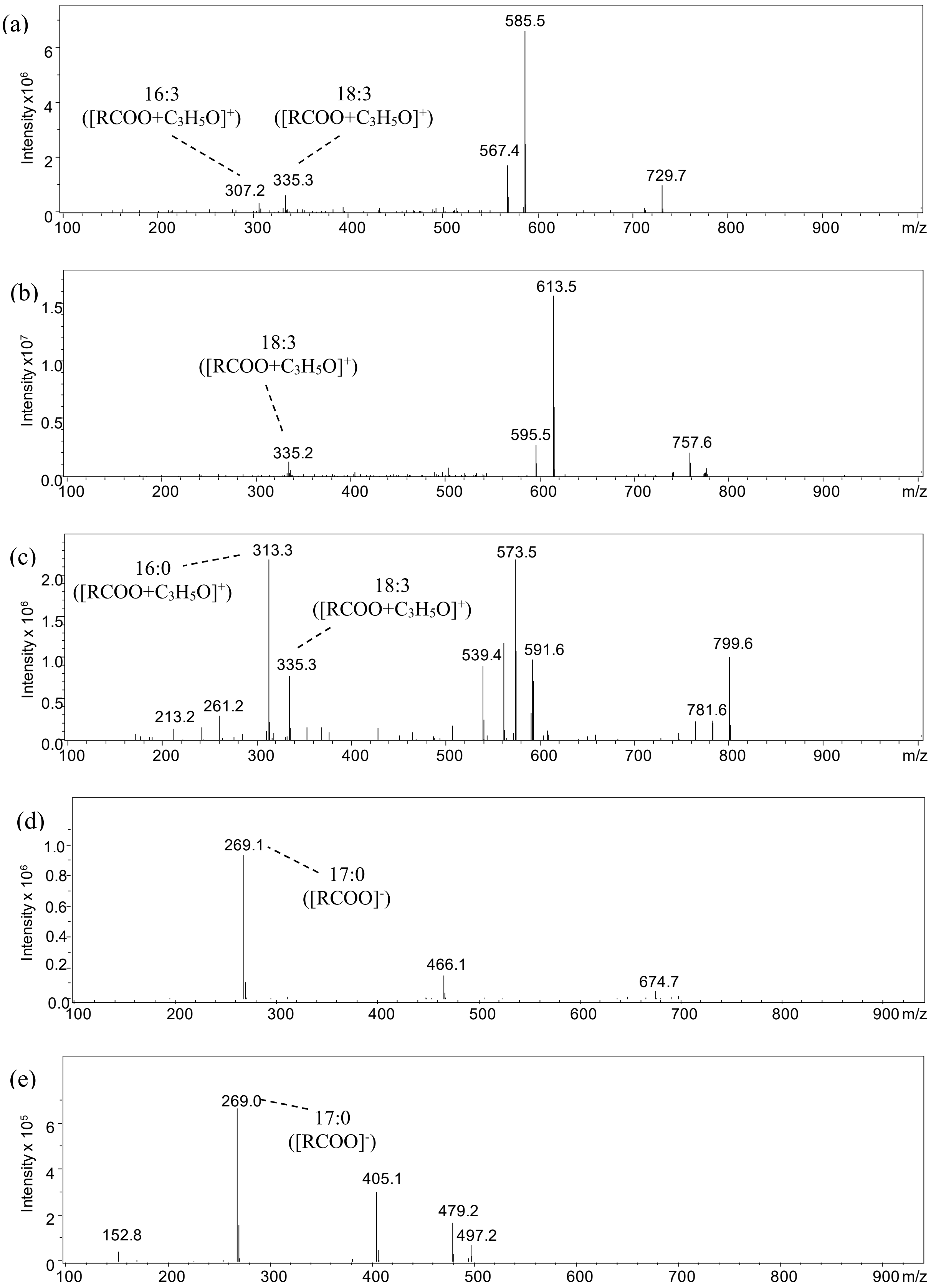
(a)
585.5
16:3
([RCOO+C3H5O]+)
18:3
([RCOO+C3H5O]+)
567.4
307.2
335.3
729.7
Intensity x10^6
m/z
(b)
613.5
18:3
([RCOO+C3H5O]+)
595.5
757.6
335.2
Intensity x10^7
m/z
(c)
313.3
573.5
16:0
([RCOO+C3H5O]+)
18:3
([RCOO+C3H5O]+)
539.4
591.6
799.6
335.3
213.2
261.2
781.6
Intensity x 10^6
m/z
(d)
269.1
17:0
([RCOO]-)
466.1
674.7
Intensity x 10^6
m/z
(e)
269.0
17:0
([RCOO]-)
405.1
479.2
497.2
152.8
Intensity x 10^5
m/z

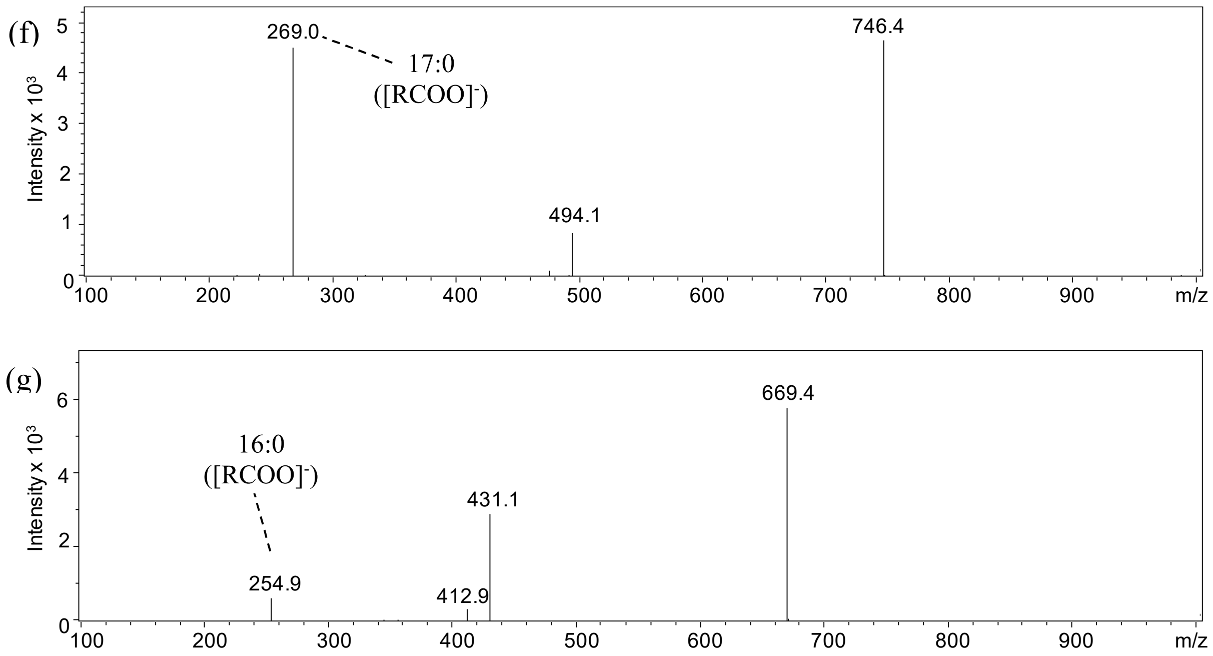

Figure S2 MS/MS fragmentation spectra for confirmation of acyl groups in polar lipid species using positive ionisation (a) MGDG 34:6,18:3/16:3($[M+NH_4]^+$=764.5 *m/z*) (b) DGDG 36:6, 18:3/18:3 ($[M+NH_4]^+$=954.5*m/z*) (c) SQDG 34:3, 16:0/18:3 ($[M+NH_4]^+$=835.5*m/z*), and negative ionisation (d) PE 34:0, 17:0/17:0 ($[M-H]^-$=719.5*m/z*) (e) PG 34:0,17:0/17:0 ($[M-H]^-$=750.5*m/z*) (f) PC 34:0, 17:0/17:0 ($[M+acetate]^-$=820.5*m/z*) and (g) DGTS 32:0, 16:0/16:0 ($[M+acetate]^-$=770.5*m/z*). Note that MS^2 and MS^3 fragmentation (data independent acquisition) was required for PC and DGTS species, respectively, to yield acyl ions. Ions corresponding to acyl fragments indicated inset spectra.
